# *In vivo* assessment of cardiac metabolism and function in the abdominal aortic banding model of compensated cardiac hypertrophy

**DOI:** 10.1093/cvr/cvv101

**Published:** 2015-03-06

**Authors:** Anne-Marie L. Seymour, Lucia Giles, Vicky Ball, Jack J. Miller, Kieran Clarke, Carolyn A. Carr, Damian J. Tyler

**Affiliations:** 1 School of Biological, Biomedical and Environmental Sciences, University of Hull, Hull HU6 7RX, UK; 2 Department of Physiology, Anatomy and Genetics, University of Oxford, Sherrington Building, Parks Road, Oxford OX1 3PT, UK

**Keywords:** Dynamic nuclear polarization, Cardiac hypertrophy, Metabolic remodelling, Pyruvate dehydrogenase, ^13^C magnetic resonance spectroscopy

## Abstract

**Aims:**

Left ventricular hypertrophy is an adaptive response of the heart to chronic mechanical overload and can lead to functional deterioration and heart failure. Changes in cardiac energy metabolism are considered as key to the hypertrophic remodelling process. The concurrence of obesity and hypertrophy has been associated with contractile dysfunction, and this work therefore aimed to investigate the *in vivo* structural, functional, and metabolic remodelling that occurs in the hypertrophied heart in the setting of a high-fat, high-sucrose, Western diet (WD).

**Methods and results:**

Following induction of cardiac hypertrophy through abdominal aortic banding, male Sprague Dawley rats were exposed to either a standard diet or a WD (containing 45% fat and 16% sucrose) for up to 14 weeks. Cardiac structural and functional characteristics were determined by CINE MRI, and *in vivo* metabolism was investigated using hyperpolarized ^13^C-labelled pyruvate. Cardiac hypertrophy was observed at all time points, irrespective of dietary manipulation, with no evidence of cardiac dysfunction. Pyruvate dehydrogenase flux was unchanged in the hypertrophied animals at any time point, but increased incorporation of the ^13^C label into lactate was observed by 9 weeks and maintained at 14 weeks, indicative of enhanced glycolysis.

**Conclusion:**

Hypertrophied hearts revealed little evidence of a switch towards increased glucose oxidation but rather an uncoupling of glycolytic metabolism from glucose oxidation. This was maintained under conditions of dietary stress provided by a WD but, at this compensated phase of hypertrophy, did not result in any contractile dysfunction.

## 1. Introduction

Heart failure continues to be a major cause of death in the Western World. Despite current therapies, the prognosis is poor and patient care is often costly. Patients with heart failure often have pre-existing hypertension and hypertrophy, recognized as independent risk factors in the development of heart failure.^1^ Left ventricular hypertrophy (LVH), the adaptive response of the heart to chronic overload, is characterized by cellular and structural remodelling,^2^ which may underpin the transition from compensated hypertrophy to decompensated heart failure. Studies using ^31^P phosphorus magnetic resonance spectroscopy (MRS) have demonstrated a marked reduction in the energy reserves in the hypertrophied heart, leading to the suggestion that the failing heart is energy depleted.^3^ In particular, the hypertrophied heart undergoes significant metabolic remodelling, switching from dependence upon fatty acids for energy provision to reliance on carbohydrates.^4,5^ This switch is underpinned by altered expression of key transcription factors including PPARα and PGC1α, which in turn cause decreased expression of the enzymes involved in fatty acid oxidation.^6^

Obesity is a growing threat to health owing to its association with a number of cardiovascular risk factors including hypertension, insulin resistance, and dyslipidaemia (metabolic syndrome).^7^ Exposure of the hypertrophied heart to surplus lipids and nutrients, as occurs in obesity, may result in further dysregulation of metabolism and energy provision.^8,9^ Ultimately, the combination of hypertrophy and obesity may accelerate the onset of contractile dysfunction, cell death, and heart failure. Studies to date have generated conflicting data in that high-lipid diets have been both beneficial and deleterious to cardiac function. An extended study using a variety of dietary manipulations including a Western diet (WD), consisting of high saturated lipid and sucrose content, demonstrated the greatest reduction in cardiac power in the WD group, as well as obesity, whereas in the high fat, low carbohydrate group, obesity was comparable, but no reduction in cardiac power occurred.^10^ However, the cellular mechanisms that underpin this deterioration in function in the heart confronted by lipid and carbohydrate overload in the setting of hypertrophy and failure are complex and incompletely understood.

Many of the experimental studies on metabolic alterations have used *in vitro* experimentation on isolated hearts, assessing metabolic fluxes with the use of ^14^C or ^13^C-labelled substrates.^11–13^ The advent of hyperpolarized magnetic resonance (MR) spectroscopy provides a powerful means to enhance the sensitivity of ^13^C MR investigations. In combination with the rapid dissolution of small ^13^C-labelled hyperpolarized substrates, this gives a novel method with which to study metabolic flux *in vivo*.^14–16^

The aim of this study was therefore to investigate the *in vivo* structural, functional, and metabolic remodelling that occurs during the time course of cardiac hypertrophic development in the setting of excess nutrient supply. In particular, the impact of a WD on the structure, function, and metabolism of the *in vivo* heart exposed to abdominal aortic banding (AAB) was determined through the use of CINE MRI and hyperpolarized ^13^C MRS to assess pyruvate dehydrogenase (PDH) flux and incorporation of pyruvate into the tricarboxylic acid (TCA) cycle. This study utilized the relatively mild model of physiological hypertrophy generated by AAB as it allowed us to explore the metabolic alterations that occurred over a period of time as hypertrophy developed. The AAB model was chosen over genetic models like the Spontaneously Hypertensive Rat (SHR), because the SHR has a loss of function mutation in the gene encoding for fatty acid translocase (FAT/CD36), a sarcolemmal fatty acid transporter that is crucial for the transport of long chain fatty acids (LCFA) across the plasma membrane. Such a mutation severely impacts the metabolic phenotype induced by cardiac hypertrophy. The AAB model was also chosen over more severe hypertrophy models, such as the transverse aortic constriction (TAC) model, as they can result in a very rapid onset of overt heart failure, which would have limited our ability to examine the metabolic changes that occur during the highly clinically relevant period of compensated hypertrophy.

## 2. Methods

[1-^13^C] and [2-^13^C]pyruvic acid were obtained from Sigma-Aldrich Company Ltd (Dorset, UK). Male Sprague Dawley rats (Body weight 200–250 g, *n* = 48) were obtained from Harlan UK. All animals were housed on a 12:12-h light–dark cycle and studies were performed between 7 a.m. and 1 p.m., during the early absorptive (fed) state. All investigations conformed to Home Office Guidance on the Operation of the Animals (Scientific Procedures) Act (HMSO) of 1986, to institutional guidelines and to the Directive 2010/63/EU of the European Parliament. A local University ethics review board also approved experiments.

### 2.1 Induction of cardiac hypertrophy

Cardiac hypertrophy was induced surgically in male Sprague Dawley rats (*n* = 24) via banding of the abdominal aorta as described previously.^11^ Anaesthesia was induced using 3% isofluorane in 3 L/min of oxygen and maintained using 2.5% isofluorane per litre O_2_. In brief, a laparotomy was performed and the aorta exposed at the level of the renal arteries. A reproducible band was placed using a 0.7 mm OD blunted needle and 3.0 mm suture where the needle was withdrawn leaving a ligature in place. Sham operated animals (*n* = 24) underwent the same procedure but without the ligation.

Animals were allowed to recover and 24–48 h post-surgery; experimental (AAB) and sham animals were divided into two groups, one that received a standard chow diet and the other that received a WD consisting of 45% saturated fat and 16% sucrose (*Table 1*). Animals were studied at 4, 9, and 14 weeks post-surgery using hyperpolarized ^13^C MR spectroscopy and CINE MRI.

**Table 1:** Dietary composition of standard and WDs

	Calorie composition (%)	
	Standard chow	WD^a^
Carbohydrates	69	35
Total protein	19	20
Total fat	11	45
Saturated fat	6	16
Monounsaturated fat	4	20
Total polyunsaturated fat	1	8

^a^Contains carbohydrate from sucrose (16%), rice, and starch.

### 2.2 MR investigations

**Figure 1:**
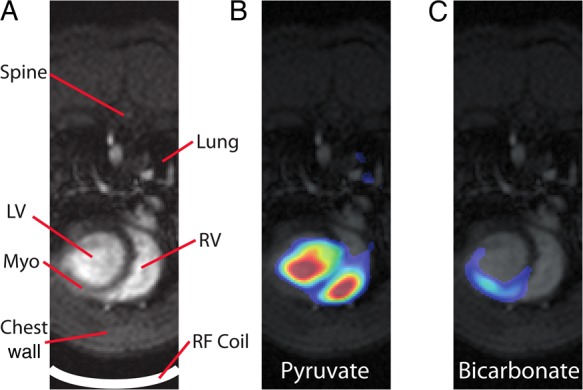
Cardiac hyperpolarized ^13^C signal localization is achieved via the high local sensitivity of the RF surface coil used and the high metabolic rate of the heart relative to other organs/tissues within the sensitive region of the coil (*A*, LV, left ventricular lumen; RV, right ventricular lumen; myo, myocardial tissue). This figure, acquired with a 3D spectral-spatial, echo planar spectroscopic imaging sequence [matrix: 32 × 32 × 12, field of view: 64 × 64 × 45.5 mm^3^, acquired resolution: 2 × 2 × 4 mm^3^, echo time (TE): 16.3 ms, repetition time (TR): 1RR interval (≈150 ms); flip angle: 17° pyruvate/61°bicarbonate], demonstrates that the pyruvate signal (*B*) originates primarily from the blood in the chambers of the heart, while the downstream metabolic signals, in this example bicarbonate (*C*), are originating from the front wall of the left ventricle.

At 4, 9, and 14 weeks post-surgery, rats received an intravenous injection of either [1-^13^C]pyruvate together with blood sampling from the saphenous vein or [2-^13^C]pyruvate together with a CINE MRI investigation to determine cardiac morphology and function. Anaesthesia was induced by 2.5–3% isofluorane in oxygen and nitrous oxide (O_2_:N_2_O 4:1, total of 2 L/min). The adequacy of anaesthesia was initially assessed based on the loss of the pedal response and was then continuously monitored through the evaluation of the heart and respiration rates. Anaesthesia was maintained by means of 2% isofluorane delivered during the experiment (O_2_:N_2_O 4:1, total of 2 L/min). A tail vein cannulation for i.v. administration of hyperpolarized solutions was performed before the animals were subsequently placed in a home-built MR animal handling system.^17^ The ECG, respiration rate, and body temperature were monitored throughout the experiment, and air heating was provided to maintain the body temperature at 37°C as previously described.^18^ A home-built ^1^H/^13^C butterfly surface coil (loop diameter, 20 mm) was placed over the rat chest, localizing the signal from the heart due to the high local sensitivity of the surface coil and the high metabolic rate of the heart (*Figure 1*). The animal was positioned with the heart at isocentre in a horizontal bore 7 T MR scanner interfaced to a direct-drive console (Varian Inc., Yarnton, UK) and correct positioning was confirmed by the acquisition of an axial proton fast low-angle shot (FLASH) image. An ECG-gated shim was used to reduce the proton line width to ∼120 Hz.^16^ Animals were allowed to recover fully from anaesthesia for serial MR investigations.

### 2.3 *In vivo* assessment of pyruvate dehydrogenase flux and oxidative metabolism

PDH flux was determined using hyperpolarized [1-^13^C]pyruvate and incorporation of pyruvate into the TCA cycle evaluated using hyperpolarized [2-^13^C]pyruvate. Hyperpolarized pyruvate was generated using ∼40 mg of either [1-^13^C]pyruvic acid or [2-^13^C]pyruvic acid doped with 15 mM trityl radical (OXO63, Oxford Instruments, Abingdon, UK) and 3 μL Dotarem (1:50 dilution, Guerbet, Birmingham, UK) in a prototype polarizer system, with 45 min of microwave irradiation as previously described.^19^ The sample was subsequently dissolved in a pressurized and heated alkaline solution, containing 2.4 g/L sodium hydroxide and 100 mg/L EDTA dipotassium salt (Sigma-Aldrich), to yield a solution of 80 mM hyperpolarized sodium [1-^13^C]pyruvate or [2-^13^C]pyruvate with a polarization of ∼30 or ∼20%, respectively, and at physiological temperature and pH.^16^

Immediately following dissolution, 1 mL of the ^13^C hyperpolarized substrate was injected intravenously into the anaesthetised animal over 10 s. Sixty individual ECG-gated ^13^C MR pulse-acquire cardiac spectra were acquired over 1 min after injection (repetition time, 1 s; excitation flip angle, 5°; sweep width, 13 593 Hz; acquired points, 2048).^16^

All cardiac ^13^C spectra were analysed using the AMARES algorithm in the jMRUI software package.^20^ Spectra were direct current offset corrected based on the last half of acquired points. For data acquired following the injection of hyperpolarized [1-^13^C]pyruvate, the peak areas of [1-^13^C]pyruvate, [1-^13^C]lactate, [1-^13^C]alanine, [^13^C]carbon dioxide (^13^CO_2_), and [^13^C]bicarbonate were quantified at each time point and used as input data for a kinetic model.^21^ The kinetic model developed for the analysis of hyperpolarized [1-^13^C]pyruvate MRS data was based on a model initially developed by Zierhut *et al.*^22^ and further developed by Atherton *et al.*^21^ The spectra acquired following injection of hyperpolarized [2-^13^C]pyruvate were summed for the first 30 s following appearance of [2-^13^C]pyruvate, to increase the sensitivity of measurements because of low signal to noise ratio. The peak areas of [1-^13^C]acetylcarnitine, [1-^13^C]citrate, and [5-^13^C]glutamate were quantified in the summed spectra and normalized to the peak area of [2-^13^C]pyruvate.

### 2.4 CINE MRI

Cardiac structure and function were determined *in vivo* by CINE MRI at 4, 9, and 14 weeks post-surgery.^23^ A 72 mm quadrature birdcage transmit/receive radio frequency coil was used to obtain MR images (Rapid Biomedical, Rimpar, Germany). Long- and short-axis scout images were acquired so that true short-axis images could be planned using a segmented, ECG-triggered FLASH sequence.^24^ CINE-MR images, consisting of 28–35 frames per heart cycle, were acquired in 7–10 contiguous slices in the short-axis orientation covering the entire heart.^18^ The imaging parameters were as follows: field of view = 51.2 × 51.2 mm, matrix size = 256 × 256, slice thickness = 1.6 mm, TE/TR = 1.43/4.6 ms, 0.5 ms/17.5° Gaussian RF excitation pulse, and 6 averages. The total experimental time was ∼50 min per animal. Heart rate remained stable throughout the procedure. End-diastolic (ED) and end-systolic (ES) frames were selected as those with the largest and smallest cavity volumes, respectively. Epicardial and endocardial borders were outlined using the freehand drawing function of ImageJ (National Institutes of Health, USA). Measurements from all slices were summed to calculate ED volume (EDV), ES volume (ESV), stroke volume (SV = EDV − ESV), ejection fraction (EF = SV/EDV), cardiac output (CO = SV × heart rate), and cardiac index (CO/body weight). LV mass was calculated as myocardial area × slice thickness × myocardial specific gravity (1.05).^18^

### 2.5 Serum analyses

Blood samples were collected from the saphenous vein at all time points immediately prior to [1-^13^C]pyruvate hyperpolarization studies. Whole blood was centrifuged to obtain plasma (15 600 g for 10 min at 4°C) and analysed for plasma metabolites [glucose, lactate, triglycerides (TAGs), low-density lipoproteins (LDLs), high-density lipoproteins (HDLs), cholesterol, and β-hydroxybutyrate] using an ABX Pentra 400 (Horiba ABX Diagnostics). At the 14-week time point, plasma insulin levels were assessed on post mortem plasma using an insulin ELISA kit (Mercodia, Sweden).

### 2.6 Tissue metabolite analyses

After the MR investigations at 14 weeks post-surgery, animals were euthanized with an overdose of isoflurane (5% isoflurane with 2 L/min oxygen), and heart tissue, abdominal fat tissue, and kidneys were harvested, weighed, and, where appropriate, freeze clamped with Wollenberger tongs cooled in liquid nitrogen. Tissues were stored at −80°C until further analysis. Tissues were then ground into a fine powder using a liquid nitrogen cooled pestle and mortar, extracted as appropriate for the assay and analysed as described below.

### 2.7 Cardiac TAG assay

TAG was extracted from ∼25–50 mg of powdered cardiac tissue following mixing and overnight suspension in 8 mL of Folch Solution (CHCl_3_:MeOH; 2:1) and 2 mL of distilled water. The aqueous phase was subsequently removed leaving the organic layer, which was evaporated to dryness and re-suspended in 1 mL of cold ethanol to form the sample solution. Approximately 100 µL of the sample solution was evaporated to dryness overnight and the sample re-suspended in 20 µL of cold ethanol from which the TAG concentration was measured spectrophotometrically using a Randox triglyceride assay kit (Randox Laboratories Ltd).

### 2.8 Western blotting

Frozen tissue was crushed and lysis buffer added before tissue was homogenized, a protein assay established the protein concentration of each lysate. The same concentration of protein from each sample was loaded on to 12.5% SDS–PAGE gels and separated by electrophoresis.^25^ A primary antibody for PDH kinase 4 (PDK4) was kindly donated by Prof. Mary Sugden (Queen Mary's, University of London, UK). Even protein loading and transfer were confirmed by Ponceau staining (0.1% w/v in 5% v/v acetic acid, Sigma-Aldrich). Bands were quantified using the UN-SCAN-IT gel software (Silk Scientific, USA) and normalized to a loading control (Cyclophilin B, Abcam). All samples were run in duplicate on separate gels to confirm results.

### 2.9 Statistical analysis

All data are presented as mean ± SD. Statistical significance of the effects of AAB and diet on the acquired data were assessed using a two-way ANOVA at each individual time point. In cases where the interaction term was significant, the differences were evaluated in more detail by separately analysing the effects of AAB and diet using a Bonferroni corrected, two-sample unpaired *t*-test assuming equal variances. Statistical significance was considered at the *P* ≤ 0.05 level.

## 3. Results

### 3.1 Structural remodelling

**Figure 2:**
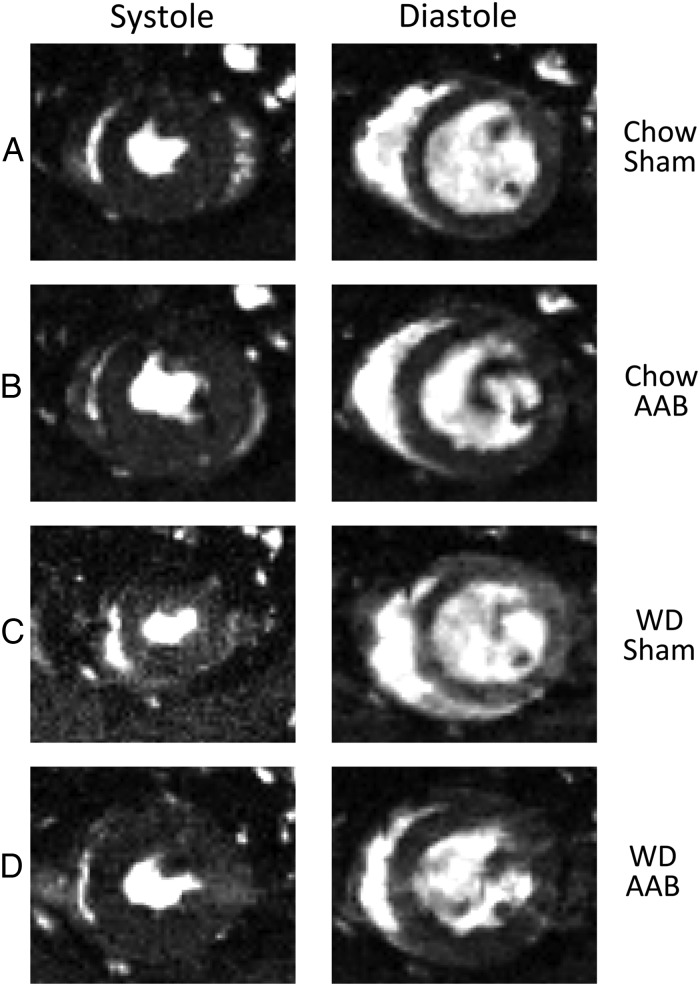
Representative *in vivo* CINE MR images of sham and AAB hearts at 14 weeks post-surgical induction of cardiac hypertrophy during systole and diastole, (*A* and *B*) on standard chow diet, (*C* and *D*) on WD.

**Figure 3:**
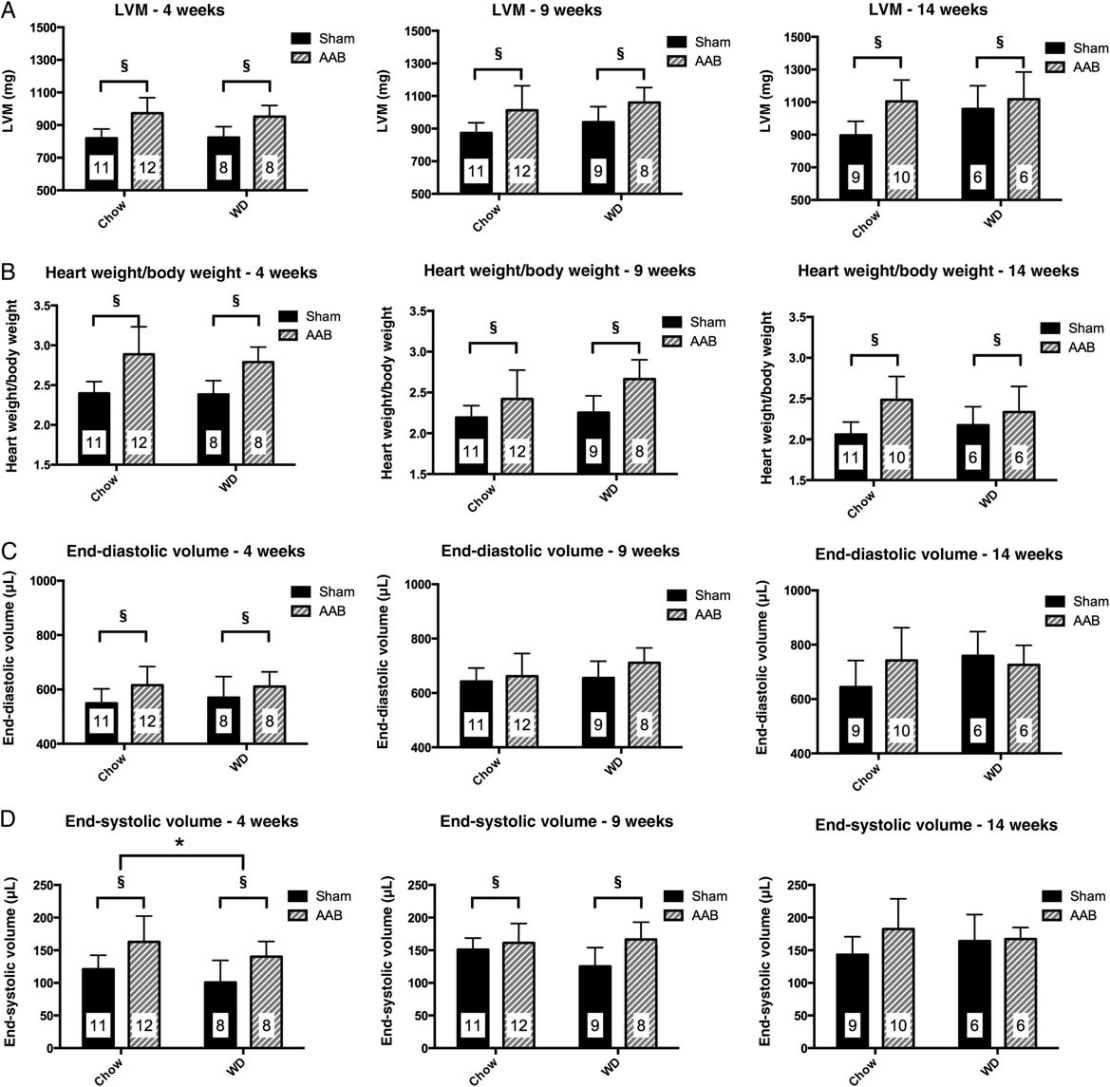
Cardiac structural parameters assessed in control and AAB animals at 4, 9, and 14 weeks post-surgical induction of cardiac hypertrophy. (*A*) LVM, (*B*) heart weight to body weight ratio, (*C*) end-diastolic volume, and (*D*) end-systolic volume. *P < 0.05 in WD groups compared with standard chow groups and ^§^*P* < 0.05 in AAB groups compared with sham control groups. Group sizes as indicated on individual bars.

*In vivo* CINE MRI was used to study the extent of structural remodelling in hearts of animals subjected to AAB over time. *Figure 2* shows representative images of hypertrophied and control hearts in systole and diastole after 14 weeks of exposure to standard chow (*Figure 2A* and *B*) or WD (*Figure 2C* and *D*) with evident enlargement of the ventricle in AAB hearts independent of any dietary modification. Quantitatively, left ventricular mass (LVM) was markedly augmented in the AAB group compared with its respective control by 4 weeks post surgery (17%) and this increase was maintained over 9 (14%) and 14 weeks (14%), irrespective of dietary manipulation (*Figure 3A*). This was further reflected in the hypertrophic index of LVM to body weight ratio (*Figure 3B*), indicating that the hypertrophy occurred primarily as a result of the hypertension induced by aortic banding rather than any effect of the diet.

In terms of body weight, there was no significant difference between AAB and control animals at 4 or 9 weeks irrespective of diet (*Table 2*). However by 14 weeks, both WD fed groups showed a significantly greater body weight than their standard chow counterparts (WD 480 ± 40 g vs. chow 440 ± 30 g), with signs of increased adiposity evidenced by larger abdominal fat deposits. This weight gain was independent of the hypertrophy present in the AAB group.

**Table 2:** *In vivo* structural and functional characteristics of hearts from control and abdominal aortic banded animals at 4, 9, and 14 weeks post induction of cardiac hypertrophy

	Sham—Chow	Sham—WD	AAB—Chow	AAB—WD
	**4 Weeks**			
*n*	11	8	10	8
Body weight/g	340 ± 20	350 ± 20	340 ± 20	340 ± 20
End-diastolic volume/μL	550 ± 50	570 ± 80	620 ± 70^§^	610 ± 50^§^
End-systolic volume/μL	120 ± 20	100 ± 30*	160 ± 40^§§§^	140 ± 20*^,§§§^
Ejection fraction/%	78 ± 4	83 ± 5**	74 ± 5^§§^	77 ± 3**^,§§^
Stroke volume/μL	430 ± 50	470 ± 50	450 ± 60	470 ± 50
Heart rate/bpm	350 ± 20	360 ± 40	360 ± 30	360 ± 30
Cardiac output/mL min^−1^	150 ± 20	170 ± 20*	160 ± 20	170 ± 20*
	**9 Weeks**			
*n*	11	9	10	8
Body weight/g	400 ± 30	420 ± 30	420 ± 20	400 ± 40
End-diastolic volume/μL	640 ± 50	660 ± 60	660 ± 80	710 ± 60
End-systolic volume/μL	150 ± 20	130 ± 30	160 ± 30^§§^	170 ± 30^§§^
Ejection fraction/%	76 ± 4	81 ± 3*	75 ± 5^§^	77 ± 4*^,§^
Stroke volume/μL	490 ± 60	530 ± 40*	500 ± 80	550 ± 60*
Heart rate/bpm	350 ± 40	380 ± 30	360 ± 30	350 ± 40
Cardiac output/mL min^−1^	170 ± 30	190 ± 30	180 ± 30	190 ± 30
	**14 Weeks**			
*n*	9	6	8	6
Body weight/g	440 ± 30	490 ± 40**	450 ± 30	480 ± 40**
End-diastolic volume/μL	640 ± 100	760 ± 90	740 ± 120	730 ± 70
End-systolic volume/μL	140 ± 30	160 ± 40	180 ± 50	170 ± 20
Ejection fraction/%	78 ± 2	79 ± 4	75 ± 5	77 ± 4
Stroke volume/μL	500 ± 70	600 ± 70	560 ± 110	560 ± 70
Heart rate/bpm	350 ± 30	350 ± 30	360 ± 40	380 ± 30
Cardiac output/mL min^−1^	180 ± 20	210 ± 20*	200 ± 30	210 ± 30*

Data expressed as mean ± SD.

*P < 0.05 for effect of diet.

**P < 0.01 for effect of diet.

***P < 0.001 for effect of diet.

^§^*P* < 0.05 for effect of AAB.

^§§^*P* < 0.01 for effect of AAB.

^§§§^*P* < 0.001 for effect of AAB.

**Figure 4:**
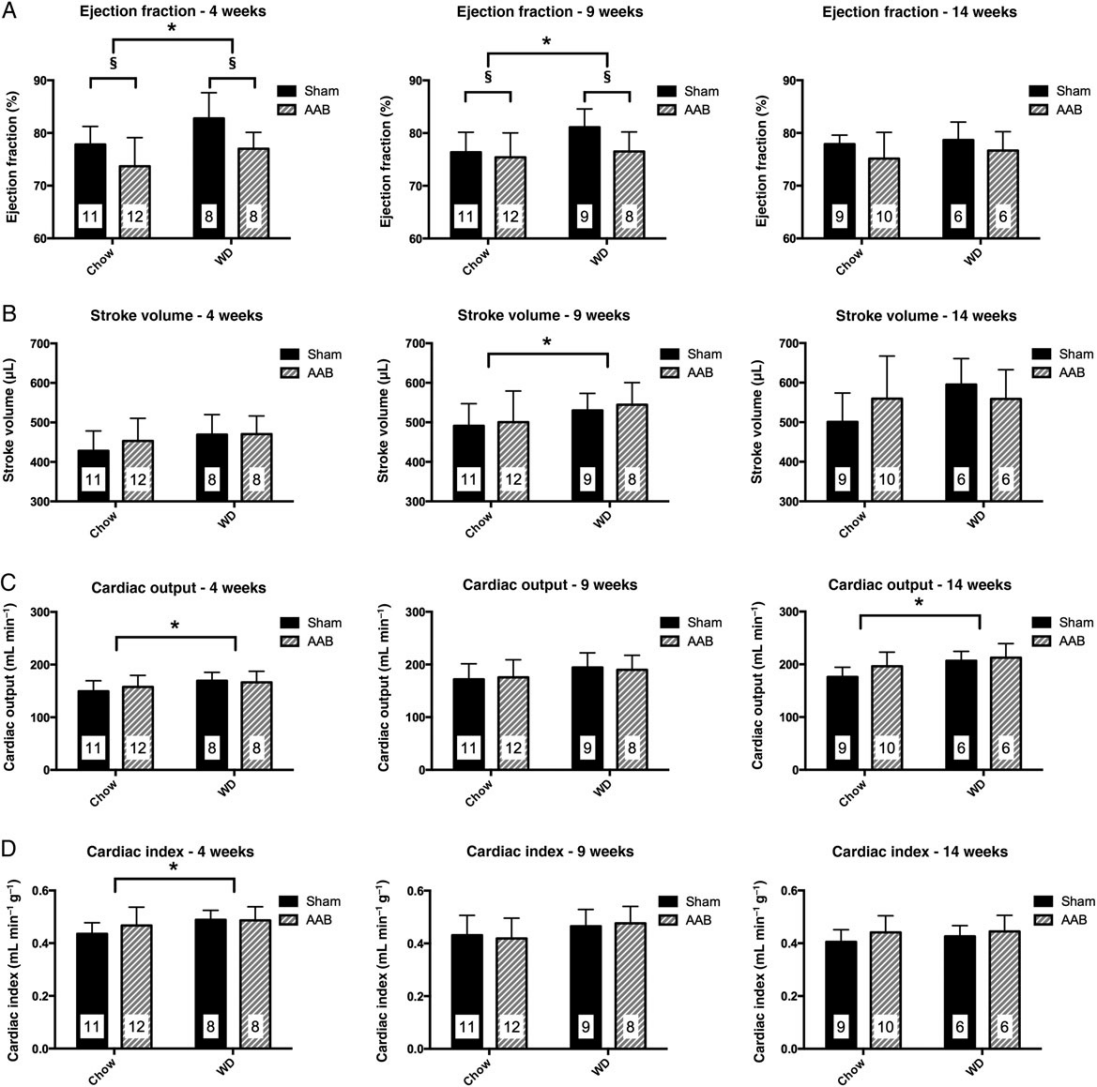
Functional characteristics of control and AAB hearts *in vivo.* (*A*) Ejection Fraction, (*B*) stroke volume, (*C*) cardiac output, and (*D*) cardiac index in sham and AAB animals at 4, 9, or 14 weeks post-surgical induction of cardiac hypertrophy, exposed to standard chow or WD. *P < 0.05 in WD groups compared with standard chow groups and ^§^*P* < 0.05 in AAB groups compared with sham control groups. Group sizes as indicated on individual bars.

Initially systolic and diastolic volumes were enlarged in AAB groups at 4 weeks post induction of hypertrophy (*Figure 3*), which resulted in a reduction in ejection fraction (*Figure 4*). However by 14 weeks, these alterations had resolved, indicating a transition from an initial acute phase of hypertrophic induction to a more compensated stage. End-systolic volumes were reduced in the WD-fed animals at the 4-week time point, resulting in an elevated ejection fraction; however, this also resolved with time and no structural differences were seen between chow and WD-fed animals at 14 weeks (*Figures 3* and *4*).

### 3.2 Functional remodelling

Functional assessment *in vivo* using MRI permitted the study of individual animals serially over the 14-week time course, which showed no indication of cardiac dysfunction in the AAB groups at any stage as reflected by the cardiac index (*Figure 4*). In addition, no effect of AAB was observed on the stroke volume or cardiac output at any time point (*Figure 4*), suggesting an enlarged yet still compliant heart in the AAB groups. Heart rate remained stable at all stages of hypertrophic development (*Table 2*) and did not differ from the sham groups, and at no stage over the time course of the experiment did the AAB animals show any signs of heart failure (breathlessness or fatigue).

In contrast, a small elevation in cardiac output was observed in both WD fed groups at 4 and 14 weeks compared with standard chow counterparts, with a trend towards increased cardiac output also visible at 9 weeks (*P* = 0.07, *Figure 4*). When normalized to body weight to generate the cardiac index (*Figure 4*), this significant elevation only remained at 4 weeks, suggesting that this was in some way related to the weight gain seen in these animals. In addition, dietary modification had no impact on heart rate (*Table 2*).

### 3.3 Metabolic remodelling

#### 3.3.1 *In vivo* assessment of PDH flux

**Figure 5:**
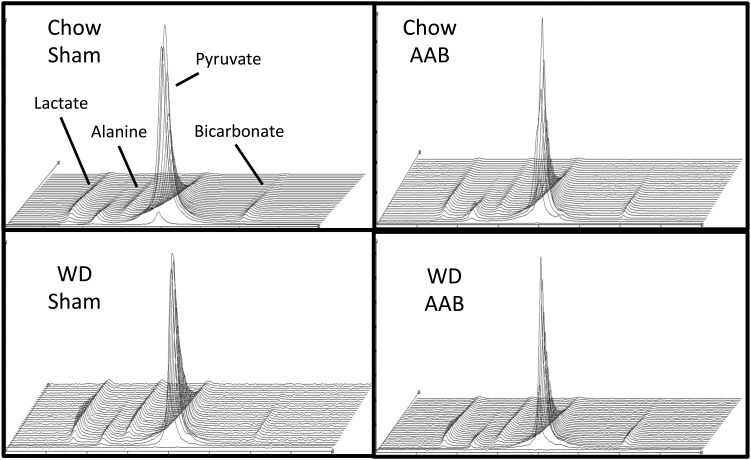
Representative *in vivo* ^13^C spectra of hearts following injection of hyperpolarized [1-^13^C] pyruvate in sham control and AAB animals at 14 weeks post-surgical induction of cardiac hypertrophy exposed to either standard chow or a WD.

**Figure 6:**
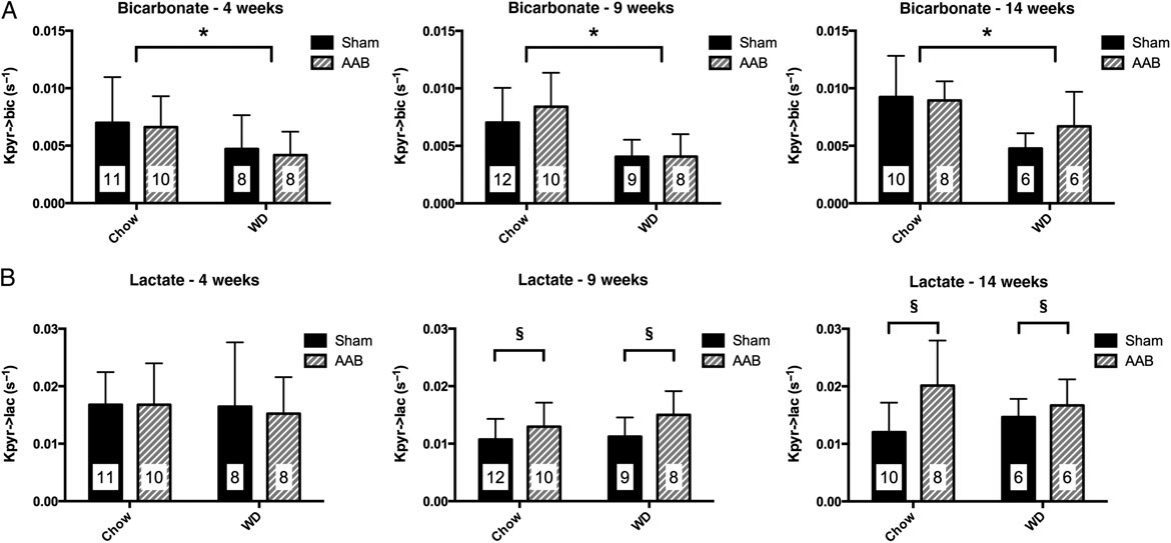
*In vivo* rates of incorporation of hyperpolarized [1-^13^C]pyruvate into (*A*) bicarbonate (pyruvate dehydrogenase flux) and (*B*) lactate at 4, 9, and 14 weeks post-surgical induction of cardiac hypertrophy and exposure to standard chow or WD. *P < 0.05 in WD groups vs. standard chow and ^§^*P* < 0.05 in AAB groups vs. respective sham controls. Group sizes as indicated on individual bars.

PDH flux was determined *in vivo* through incorporation of the hyperpolarized ^13^C label from [1-^13^C]pyruvate into bicarbonate as previously demonstrated^21^ (*Figure 5*). Rates of ^13^C label incorporation into bicarbonate in AAB groups at the different time points were not significantly different from their respective controls (*Figure 6A*), implying little effect of AAB on PDH flux *in vivo* and thus glucose oxidation over the time course of hypertrophic development. At all time points however, dietary manipulation resulted in a marked reduction in ^13^C label incorporation into bicarbonate, indicating that PDH flux was reduced in the WD groups compared with their respective standard chow groups as would be predicted from the Randle cycle.^26^

Label incorporation into alanine was unaffected by either AAB or dietary manipulation (data not shown). However, the rate of ^13^C label incorporation into lactate was markedly enhanced in AAB groups by 9 weeks and sustained by 14 weeks (*Figure 6B*), independent of diet. These findings highlight that metabolic remodelling during the progression of cardiac hypertrophy is a continuous process with an increase in glycolytic metabolism as compensated hypertrophy develops.

#### 3.3.2 *In vivo* assessment of TCA cycle metabolites

**Figure 7:**
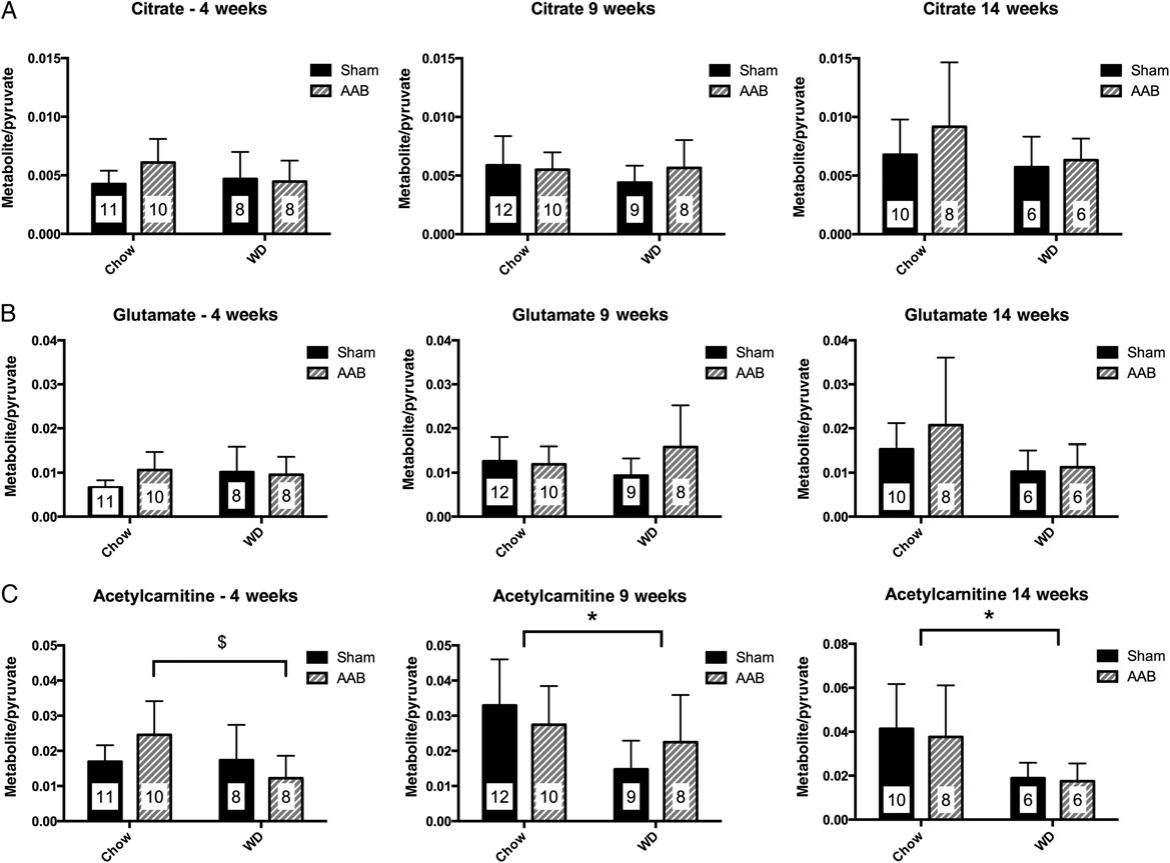
Ratio of (*A*) citrate, (*B*) glutamate, and (*C*) acetylcarnitine to injected hyperpolarized [2-^13^C]pyruvate at 4, 9, and 14 weeks post-surgical induction of cardiac hypertrophy and exposure to standard chow or WD. ^$^*P* < 0.05 WD AAB group compared with chow AAB group and *P < 0.05 WD groups compared with standard chow groups. Group sizes as indicated on individual bars.

Hyperpolarized [2-^13^C]pyruvate was used to determine incorporation of pyruvate into TCA cycle intermediates. Within the AAB groups, there was no change in the [^13^C]metabolite:[2-^13^C]pyruvate ratio for either citrate or glutamate, independent of diet (*Figure 7A* and *B*). Given that PDH flux was unaltered in the hypertrophied hearts, these observations would indicate that there was no increase in the flux of glucose into the TCA cycle during this phase of hypertrophic development. These findings are thus suggestive of a compensatory phase of cardiac hypertrophy with no adverse impact from the high-fat, high-sucrose content of the WD.

However, there was a substantial reduction in the ratio of [1-^13^C]acetylcarnitine/[2-^13^C]pyruvate within the WD-fed groups (*Figure 7C*). Previous findings have proposed a role for glycolytically derived acetylcarnitine to sustain and buffer the mitochondrial pool of acetyl-CoA, and therefore, a reduction in ^13^C label incorporation may indicate a reduced buffering capacity driven by enhanced utilization of the available carnitine for mitochondrial fatty acid transport.^27^

### 3.4 Serum metabolite levels

Serum metabolite concentrations are summarized in *Table 3*. Few differences were observed between AAB and their respective control groups at 4, 9, or 14 weeks, identifying the fact that AAB and its associated hypertension *per se* does not influence serum nutrient levels. However, as might be anticipated, dietary manipulation did have a significant impact, with animals subjected to WD feeding repeatedly exhibiting elevated plasma TAG and LDL levels and reduced plasma lactate levels. Plasma insulin levels were also assessed at the 14-week time point (*Table 3*) and showed a significant elevation in the WD-fed groups irrespective of AAB, indicating a level of insulin resistance induced by the WD.

**Table 3:** Profile of serum metabolites from control and abdominal aortic banded animals at 4, 9, and 14 weeks post induction of cardiac hypertrophy

	Sham—Chow	Sham—WD	AAB—Chow	AAB—WD
	**4 Weeks**			
*n*	10	9	8	10
Glucose/mM	12 ± 2	12 ± 2	13 ± 3	12 ± 4
Lactate/mM	2.0 ± 0.5	1.7 ± 0.4**	2.2 ± 0.3	1.6 ± 0.4**
TAG/mM	1.2 ± 0.4	2.0 ± 0.5****	1.2 ± 0.3	2.0 ± 0.7****
LDL/mM	0.4 ± 0.1	0.5 ± 0.1**	0.4 ± 0.1	0.6 ± 0.1**
HDL/mM	0.8 ± 0.2	0.9 ± 0.1	0.8 ± 0.1	0.9 ± 0.1
Cholesterol/mM	2.2 ± 0.3	2.5 ± 0.5	2.4 ± 0.4	2.7 ± 0.6
β-Hydroxybutyrate/mM	0.07 ± 0.04	0.07 ± 0.04	0.09 ± 0.05	0.1 ± 0.1
	**9 Weeks**			
*n*	8	6	8	7
Glucose/mM	16 ± 1	16 ± 1	16 ± 2	17 ± 3
Lactate/mM	2.2 ± 0.5	2.0 ± 0.5	2.3 ± 0.4	2.2 ± 0.6
TAG/mM	1.6 ± 0.6	1.9 ± 0.5*	1.5 ± 0.3	1.8 ± 0.3*
LDL/mM	0.4 ± 0.1	0.4 ± 0.2	0.4 ± 0.1	0.5 ± 0.2
HDL/mM	0.8 ± 0.2	0.8 ± 0.1	0.9 ± 0.2	0.9 ± 0.2
Cholesterol/mM	3.1 ± 0.4	3.1 ± 0.1	3.2 ± 0.3	3.4 ± 0.4
β-Hydroxybutyrate/mM	0.05 ± 0.01	0.07 ± 0.04	0.06 ± 0.02	0.07 ± 0.04
	**14 Weeks**			
*n*	8	6	7	8
Glucose/mM	14 ± 4	14 ± 3	15 ± 3	13.6 ± 0.7
Lactate/mM	2.4 ± 0.4	1.9 ± 0.5*	2.3 ± 0.3	1.8 ± 0.3*
TAG/mM	1.4 ± 0.2	1.5 ± 0.5	1.3 ± 0.4	1.7 ± 0.2
LDL/mM	0.43 ± 0.06	0.6 ± 0.2**	0.5 ± 0.1	0.7 ± 0.1**
HDL/mM	0.9 ± 0.2	0.9 ± 0.2	0.9 ± 0.2	1.03 ± 0.06
Cholesterol/mM	3.0 ± 0.8	2.7 ± 0.6	3.3 ± 0.7	3.1 ± 0.3
β-Hydroxybutyrate/mM	0.07 ± 0.02	0.08 ± 0.04	0.08 ± 0.03	0.08 ± 0.04
Insulin/μg mL^−1^	16 ± 2	30 ± 10**	15 ± 3	50 ± 30**

Data expressed as mean ± SD.

*P < 0.05 for effect of diet.

**P < 0.01 for effect of diet.

****P < 0.0001 for effect of diet.

### 3.5 PDK4 expression and cardiac TAG concentrations

**Figure 8:**
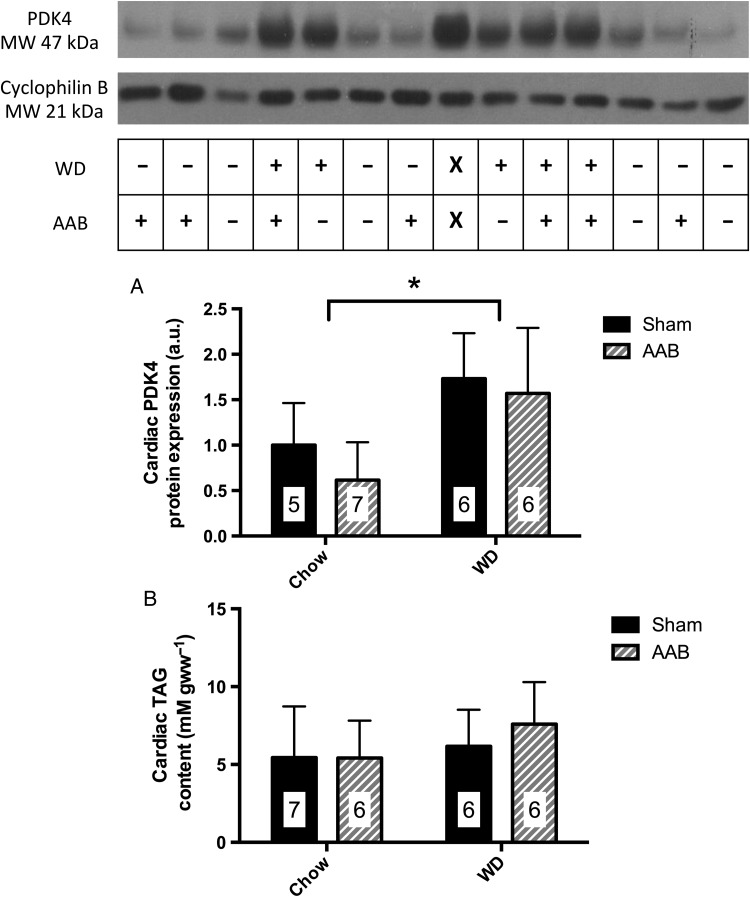
(*A*) Cardiac PDK4 protein expression and (*B*) cardiac TAG content at 14 weeks post-surgical induction of cardiac hypertrophy (AAB) and exposure to standard chow or WD. *P < 0.05 WD groups compared with standard chow groups. Group sizes as indicated on individual bars. X represents an internal standard on the western blot.

*In vitro* analysis of the expression of the key inhibitory enzyme PDK4 at the 14-week time point reflected the *in vivo* hyperpolarized ^13^C MR findings with no alteration in expression in AAB or control groups within the same dietary group but a marked elevation of PDK4 expression in WD groups (*Figure 8A*). *In vitro* analysis of cardiac TAG levels demonstrated that they were unaffected by either AAB or the WD (*Figure 8B*), indicating no elevated deposition of lipids in the cardiac tissue.

## 4. Discussion

Using the novel, non-invasive technique of hyperpolarized MRS, this *in vivo* study has investigated the temporal metabolic, structural, and functional changes that are associated with the development of cardiac hypertrophy in the setting of excess nutrient supply. AAB caused a mild hypertrophic response in the heart without any functional detriment, a response that was unaffected by dietary manipulation. Although there was clear evidence of structural remodelling as early as 4 weeks post surgical induction of cardiac hypertrophy, the metabolic alterations that developed with the progression of hypertrophy determined by hyperpolarized ^13^C MR were not observed until 9 weeks and highlighted a switch towards increased glycolytic metabolism rather than towards enhanced glucose oxidation.

### 4.1 Structural and functional impact of AAB

CINE MRI is a dynamic technique that allows measurement of both structural remodelling and cardiac function *in vivo*. The findings have clearly shown significant structural alterations in the left ventricle of AAB hearts. In line with the aim of this work to explore the structural, functional, and metabolic remodelling that occurs during the time course of cardiac hypertrophic development, the degree of hypertrophy induced in this model (14–17%) was smaller than that produced by the more severe ascending aortic constriction (30–45%),^28,29^ TAC (33%),^30^ and SHR (56%)^15^ models of cardiac hypertrophy, which more rapidly progress to heart failure but the degree of hypertrophy was clearly evident both in the increased cardiac mass as well as the initially enlarged ventricular volumes and was consistent with previous studies employing this model.^11,31^ The development of hypertrophy did not appear to be affected by the alteration in diet, although the animals maintained on the WD did show a greater weight gain by 14 weeks. This was primarily a result of increased fat mass, but the weight gain was small and could not be considered as representative of an obese model.

Although there was an increase in both end-systolic and end-diastolic dimensions in the AAB group at 4 weeks post surgery, resulting in a reduced ejection fraction, this appeared to be a transient event. These alterations may reflect the acute response of the heart to the hypertrophic pressure overload stimulus, which resolved into a compensated phase over the subsequent 10 weeks. *In vitro* studies of this model have shown early expression of ANF, a marker of hypertrophy, within a week of AAB but no evidence of heart failure at this phase.^31^ Over time, there is little evidence of functional impairment in the AAB groups suggesting development of a compensated model of LVH by 14 weeks. This does not preclude the possibility of diastolic dysfunction, which we were unable to assess in this study. It is possible that the study of alterations in diastolic function may offer a more sensitive way to explore the effects of AAB within the setting of excess nutrient supply.

### 4.2 Metabolic remodelling with the development of hypertrophy

Metabolic remodelling is a frequently observed feature of cardiac hypertrophy and heart failure. Many studies have identified a switch from fatty acid oxidation towards a reliance on glucose metabolism for energy generation in the hypertrophied and failing heart.^11,31–33^ However, the majority of these studies have used *in vitro* systems in the isolated perfused heart with defined substrate concentrations in crystalloid buffers. In this *in vivo* study, the heart has been exposed to the natural composition of metabolites within the blood—giving a more biologically relevant picture of metabolic remodelling and the functional consequences.

Here we have used the novel *in vivo* method of hyperpolarized MRS to probe the remodelling of pyruvate metabolism in the hypertrophied heart exposed to a nutrient-rich environment over time. Our findings have identified an apparent uncoupling of glucose metabolism as an early event in hypertrophic development. Throughout the time course of the experiment, no change in flux through PDH was observed, which is in contrast to single time point studies on the SHR model where an 85% increase in PDH flux was observed.^15^ The studies on the SHR model have shown a marked increase in PDH flux, supported by *in vitro* assay measurements of PDH activity. In part, the differences observed between these models could be due to a lesser degree of hypertrophic remodelling in the AAB model here compared with the SHR model or may imply that the increased reliance on glucose oxidation is a later event in the progression of hypertrophy. In addition, the SHR model is known to have multiple genetic mutations that contribute to the hypertensive phenotype.^15^ A major mutation is a loss of the sarcolemmal fatty acid transporter, FAT/CD36, which is likely to lead to a reduction in fatty acid oxidation and a consequential increase in glucose oxidation and PDH flux. It is therefore highly likely that the metabolic alterations seen in the previously study^15^ were impacted by this lack of FAT/CD36 and the ability to draw conclusions on the balance between the cause and effect of the hypertension in the light of these genetic changes is extremely challenging.

Previous studies measuring PDH activity in tissue extracts from the AAB model have identified a 69% reduction in the percentage of PDH in the active fraction.^34^ However, the hyperpolarized MRS method is unique in measuring *in vivo* flux through PDH in contrast to *in vitro* assay systems, which only assess the proportion of enzyme in the active form.

Although there was no alteration in PDH flux *in vivo*, we have observed an elevation in glycolytic flux by 9 weeks as detected by greater incorporation of the hyperpolarized ^13^C label into lactate. The level of enhanced label incorporation into lactate (26% at 9 weeks and 30% at 14 weeks) was smaller than that seen in previous hyperpolarized MRS studies investigating cancer treatment (lactate signal levels 50% higher in untreated vs. treated tumours)^35^ or acute ischaemia (lactate signal levels 138% higher in ischaemic hearts vs. control hearts)^36^ but when combined with the lack of alteration in PDH flux, it would suggest that, beyond the initial acute response to the hypertrophic stimulus, there was a degree of uncoupling of glucose oxidation from glycolysis. Lopaschuk and colleagues^37^ have suggested that this may occur as a compensatory response under conditions such as ischaemia or more chronic situations such as hypertrophy. Enhanced [1-^13^C]lactate generation was still observed at the 14-week time point suggesting, in the absence of any functional deterioration, that the observed level of glycolytic uncoupling was not a significantly deleterious adaptation. However, this does not preclude this uncoupling from becoming a detrimental factor as the LVH progresses into heart failure.

### 4.3 The effects of dietary modification

The application of a WD appears to have had little impact on cardiac structure, suggesting that, unlike other studies,^10,38,39^ exposure to a WD for this time period does not induce hypertrophy. Functionally, the WD led to a small but significant increase in stroke volume and cardiac output. Therefore, at this stage, the WD does not cause any cardiac dysfunction but rather a small degree of hyperfunction.

Further, the combination of cardiac hypertrophy with dietary manipulation using a WD does not appear to have caused any functional deterioration, consistent with other studies employing high-fat/high-sugar diets.^40,41^ However, controversy exists in this area, as augmenting the percentage of saturated fats in the diet without affecting the sugar content does appear to result in functional impairment. In these instances, studies have used up to 60% fat rather than the 45% used here. The excess supply of fat under conditions where fatty acid oxidation may be reduced, as in LVH, could result in lipotoxicity with inappropriate accumulation of lipids within the heart and cell death. Some evidence exists for this from work on isolated cardiomyocytes, which demonstrated that when palmitate was included as the fatty acid substrate it resulted in enhanced cell death as opposed to oleate that protected against apoptosis and lipotoxicity.^42^

Dietary manipulation had a major impact on PDH flux *in vivo* with an increased fat and carbohydrate composition in the diet, leading to a significant reduction in the utilization of glycolytically derived pyruvate. Although predicted from *in vitro* experimentation,^26^ hyperpolarized MRS offers a unique way to visualize this effect *in vivo*. No concomitant change in [1-^13^C]lactate production was observed with the dietary-induced decrease in PDH flux. Elevated insulin levels were observed in the WD-fed groups at the 14-week time point, which when coupled with the unaltered glucose levels, indicates a degree of insulin resistance induced by the high-fat, high-sugar diet.

Label incorporation into acetylcarnitine was markedly reduced in the WD-fed animals from 4 weeks onwards providing early evidence of the effect of dietary composition on substrate metabolism and storage. Given the role of carnitine as a co-factor in mitochondrial fatty acid transport,^32^ this suggests that carnitine availability has become limiting following enhanced fatty acid utilization promoted by an increase in dietary fatty acid availability. The implications of a reduction in the buffering of pyruvate-derived acetyl-CoA into acetylcarnitine remains unclear at this point but does not appear to affect the development of hypertrophy nor have any deleterious effect on normal function.

## 5. Conclusions

This *in vivo* study examining the metabolic alterations that occur during the development of compensatory hypertrophy suggests a switch towards increased glycolytic metabolism rather than enhanced glucose oxidation as previously described. This appears to be irrespective of dietary manipulation with a WD, which despite causing a significant reduction in flux through PDH and evidence suggesting promoted fatty acid utilization, did not correlate with any structural or functional deterioration in the heart.

**Conflict of interest:** none declared.

## Funding

This work was supported by the British Heart Foundation in the form of an Intermediate Basic Science Research Fellowship (FS/10/002/28078), a Senior Basic Science Research Fellowship (FS/14/17/30634) and a 4-year PhD Studentship. This work was also supported by EPSRC in the form of a 4-year PhD studentship, and GE Healthcare in the form of equipment support. Funding to pay the Open Access publication charges for this article was provided by the British Heart Foundation.
